# Characterization of *Mycobacterium leprae* Genotypes in China—Identification of a New Polymorphism C251T in the 16S rRNA Gene

**DOI:** 10.1371/journal.pone.0133268

**Published:** 2015-07-21

**Authors:** Youhua Yuan, Yan Wen, Yuangang You, Yan Xing, Huanying Li, Xiaoman Weng, Nan Wu, Shuang Liu, Shanshan Zhang, Wenhong Zhang, Ying Zhang

**Affiliations:** 1 Key Laboratory of Medical Virology, Department of Infectious Diseases, Huashan Hospital, Shanghai Medical College, Fudan University, Shanghai, China; 2 Beijing Tropical Medicine Research Institute, Beijing Friendship Hospital, Capital Medical University, Beijing, China; 3 Department of Molecular Microbiology and Immunology, Bloomberg School of Public Health, Johns Hopkins University, Baltimore, Maryland, United States of America; The University of Hong Kong, HONG KONG

## Abstract

Leprosy continues to be prevalent in some mountainous regions of China, and genotypes of leprosy strains endemic to the country are not known. *Mycobacterium lepromatosis* is a new species that was discovered in Mexico in 2008, and it remains unclear whether this species exists in China. Here, we conducted PCR- restriction fragment length polymorphism (RFLP) analysis to classify genotypes of 85 DNA samples collected from patients from 18 different provinces. All 171 DNA samples from skin biopsies of leprosy patients were tested for the presence of *Mycobacterium leprae* and *Mycobacterium lepromatosis* by amplifying the 16S rRNA gene using nested PCR, followed by DNA sequencing. The new species *M*. *lepromatosis* was not found among the 171 specimens from leprosy patients in 22 provinces in China. However, we found three SNP genotypes among 85 leprosy patients. A mutation at C251T in the 16S rRNA gene was found in 76% of the strains. We also found that the strains that showed the 16S rRNA C251T mutation belonged to SNP type 3, whereas strains without the point mutation belonged to SNP type 1. The SNP type 3 leprosy strains were observed in patients from both the inner and coastal regions of China, but the SNP type 1 strains were focused only in the coastal region. This indicated that the SNP type 3 leprosy strains were more prevalent than the SNP type 1 strains in China. In addition, the 16S rRNA gene sequence mutation at C251T also indicated a difference in the geographical distribution of the strains. To our knowledge, this is the first report of a new polymorphism in 16S rRNA gene in *M*. *leprae* in China. Our findings shed light on the prevalent genotypes and provide insight about leprosy transmission that are important for leprosy control in China.

## Introduction

Leprosy is a chronic infectious disease caused by *Mycobacterium leprae* [1]. The infection can lead to skin lesions, severe nerve damage and disabilities. Although the leprosy epidemic has been recorded since the time of Confucius (551–479 BCE), how leprosy spread into and within China has not been clearly elucidated [2]. Moreover, few studies on leprosy genotypes have been conducted within regional areas [3], and they could not explain the general epidemic status of leprosy in China. Therefore, it is necessary to determine the genotypic features and geographic distribution of Chinese leprosy strains. A comparison with strain genotypes from Asia and worldwide can shed light on leprosy transmission in China, and further analyses can help predict future trends.

Whole genome sequences for four strains of *M*. *leprae* from Brazil, India, Thailand, and the USA have been completed [4], and comparative genomic and phylogeographic analyses of these strains revealed that, based on single nucleotide polymorphisms (SNPs) within the genomes, there are four genotypes around the world, and that parallel dominant epidemic SNP genotypes exist in different geographic regions. Thus, SNP genotyping of leprosy strains can be used to search for evidence of human migration and leprosy transmission in the world.

The 16S rRNA gene is the most conserved gene across all mycobacterial species. Consequently, 16S rRNA sequence analysis can be used as a tool to identify different species of mycobacteria [5]. A new leprosy-causing species called *Mycobacterium lepromatosis* was discovered in Mexico in 2008 [6]. This pathogen was subsequently found in Singapore and more recently in Canada, Brazil, Malaysia, Myanmar, Uganda as well as in other countries worldwide [7–9]. Further analysis of 22,814 nucleotides from 20 genes revealed a 9.1% difference between the new and old leprosy pathogens to substantiate a species-level divergence, which occurred approximately 10 million years ago [10]. The 9.1% sequence difference contrasts sharply with the 0.005% difference uncovered by genome sequencing and multi-locus genotyping between *M*. *leprae* strains worldwide. To explore whether this novel leprosy pathogen is present in China, we analyzed 171 leprosy skin biopsy tissue samples from patients in different provinces of China by using nested PCR [11]. In addition, we analyzed the SNP genotype features and their relationship to a newly identified 16S rRNA gene polymorphism to determine the prevalent genotypes in the country.

## Materials and Methods

### Ethics Statement

This study was approved by the Ethics Committee of the Beijing Tropical Medicine Research Institute. Written informed consent was obtained from the participants, and if the patients were under 18 years of age, their parents provided written informed consent. The skin biopsy tissues of leprosy patients and relevant information, including age, sex, and ethnicity and clinical features of the disease were collected.

### Samples

One hundred and seventy-one skin biopsy tissue samples from leprosy patients were collected, and DNA was extracted using the QIAamp DNA Mini Kit (Qiagen). These leprosy patient specimens from 22 Chinese provinces were preserved at the Beijing Institute of Tropical Diseases. Leprosy clinical types were classified according to Ridley-Jopling system into lepromatous leprosy (LL), borderline (BB), borderline lepromatous (BL), borderline tuberculoid (BT), tuberculoid (TT). The general demographic characteristics of these samples are shown in Table 1.

**Table 1:** General demographic characteristics of the leprosy specimens.

Clinical type*	Sex		Age			Ethnicity					
	Male	Female	Range	Mean	Median	Han	Zhuang	Zang	Miao	Tujia	0thers
LL	57	23	22–77	48.7	49	45	1	6	1	2	25
BL	31	22	30–85	49.9	51	23	1	0	2	1	26
BB	4	3	16–57	42	40	5	0	0	0	0	2
BT	9	7	16–62	41.2	32	8	1	0	0	0	7
TT	5	1	18–70	41.5	31	4	2	0	0	0	0
Others	7	2	16–51	36	30	5	4	0	0	0	0
Total	113	58	16–85	47.3	47	90	9	6	3	3	60

• LL: Lepromatous leprosy; BL: borderline lepromatous; BB: Borderline; BT: Borderline tuberculoid (BT); TT: tuberculoid

### SNP typing of *M*. *leprae*

SNP types (1–4) were analyzed for the studied samples (n = 85). A PCR restriction fragment length polymorphism (RFLP)-based procedure was used for differentiation of SNP types (1–4) as described by Sakamuri et al. [12]. Sequencing of SNPs was performed for samples that could not be typed using the PCR-RFLP assay [13].

### Nested PCR and sequencing for conserved mycobacterial 16S rRNA gene

The 16S rRNA gene was amplified and sequenced as described by Han et al.[11]. In brief, two rounds of hemi-nested PCR were used to maximize detection sensitivity; the first-round PCR used primers AFBFO (5′-GCGTGCTTAACACATGCAAGTC-3′) and MLERE (5′-CTACCGTCAATCCGAGAAAACC-3′), which are common to all mycobacterial species (approximately 150 species). The resulting amplicons, 447 base pairs (bp) in size and usually faint or not easily detectable, were diluted 100-fold and further amplified by two separate second-round PCRs using primers MLERE and LPMF3 (5′-GGTCTCTTAATACTTAAACCTATTAA-3′) for *M*. *lepromatosis* (417 bp) and primers MLERE and LERF3 (5′-CTAAAAAATCTTTTTTAGTACTC-3′) for *M*. *leprae* (411 bp). The cycle parameters had the following steps: 98°C for 2 min followed by 35 cycles of denaturation (98°C for 20 sec), primer annealing (58°C, 20 sec for first-round PCR or 50°C 20 sec for second-round PCR) and extension (72°C, 40 sec), and final extension at 72°C for 5 min. A PCR premix (code RR902. lot no. A1401A. Takara) was used for the PCR reactions in a total volume of 50 μL containing 50 ng genomic DNA, 25 μL 2× premix, and 10 pmol of each primer. The target amplicons were examined by 1% agarose gel electrophoresis and the PCR products showing visible bands were gel purified and sequenced directly by Sanger sequencing. The PCR products were cloned in TA vector and sequenced by M13 primer if the samples were suspected to have mixed infection with *M*. *leprae* and *M*. *lepromatosis*.

### Statistical analysis

The SPSS 20.0 statistical software package was used for statistical analysis. The SNP genotype and 16S rRNA gene mutation percentage was compared between groups using the Pearson Chi-square test or Fisher’s exact method when possible. *P* values less than 0.05 were considered statistically significant.

## Results

### Comparative analysis of 16S rRNA and SNP genotype between leprosy patients with different pathologies

The 171 biopsy samples were subjected to nested PCR amplification and DNA sequencing for the 16S rRNA gene as described in the Methods section. BLAST analysis revealed that all sample sequences were 99% similar to Br4923 *M*. *leprae* and TN strain, and the new species *M*. *lepromatosis* sequence could not be identified from these specimens. In addition, three SNP genotypes were found among 85 samples. The majority of the leprosy strains were found to belong to SNP type 3, accounting for 78.8% (67/85) of the strains. Some leprosy strains belonged to SNP type 1, accounting for 20% of the strains (17/85). There was only one strain that belonged to SNP type 2, accounting for 1.2% (1/85) of all strains. None of the strains were found to be of SNP type 4 (Table 2).

**Table 2:** Comparison of 16S rRNA mutation and SNP genotypes in leprosy patients with different lesion pathology.

Clinical Type	16S rRNA sequence mutation			SNP genotype (%)			
	Number	C251T	%	Number	Type 1	Type 2	Type 3
LL	80	63	78.8	37	7(18.9)	1(2.7)	29(78.4)
BL	53	40	75	31	6(16.7)		25(81.3)
BB	7	6	85.7	4	1(25)		3(75)
BT	16	10	62.5	8	2(25)		6(75)
TT	6	4	66.7	2	0		2(100)
Others	9	7	77.8	3	1(33.3)		2(66.7)
Total	171	130	76.0	85	17(20)	1(1.2)	67(78.8)
Statistical tests	*x* ^*2*^	2.481		3.053			
	*p*	0.779		0.98			

**Fig 1:**
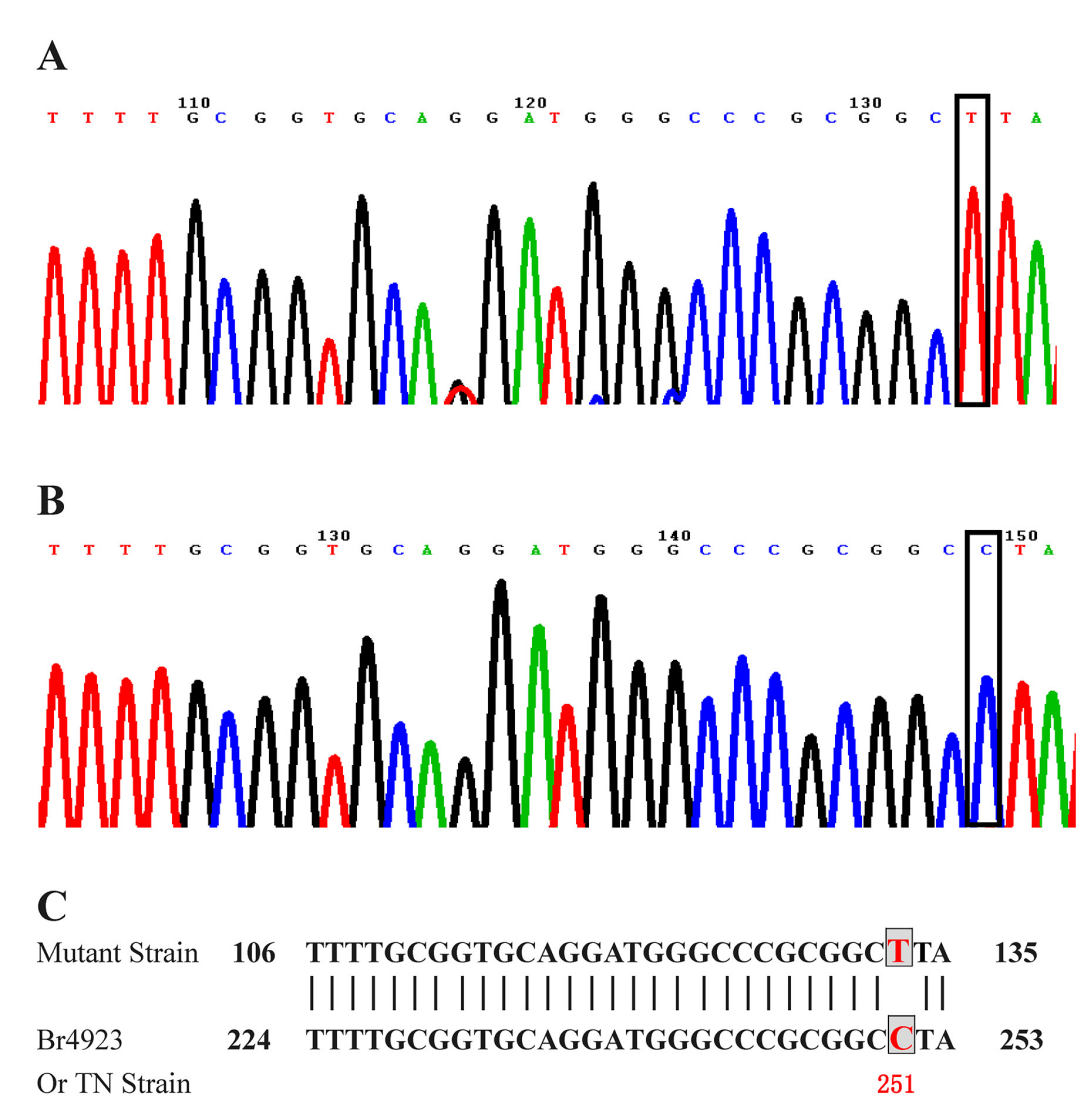
C251T polymorphism in the 16S rRNA gene of *M*. *leprae*. A, B: Chromatogram showing the C251T polymorphism in the 16S rRNA gene of mutant (A) and wildtype (B) strains. Partial sequence alignment of mutant and wildtype 16S rRNA sequence from Br4923 and TN *M*. *leprae* strains (C).

We compared the Br4923 and TN *M*. *leprae* strain using BLAST and found that 132 of 171 samples showed a mutation change of C to T located at the 251^st^ bp in the 447 bp fragment of the 16S rRNA gene (Fig 1). There was no significant difference in the 16S rRNA gene C251T polymorphism among different clinical pathological types of leprosy. There was also no significant difference between SNP genotypes and different clinical pathological types of leprosy (Table 2).

### Comparison between 16S rRNA gene mutation and SNP genotype

Comparing the 16S rRNA gene mutation with the SNP genotypes, we found that most of the 85 strains analyzed had the C251T mutation and belonged to SNP type 3 (54/67). However, the strains without the point mutation belonged to SNP type 1 (16/17) (Table 3). The mutation frequency of the 16S rRNA gene for SNP type 3 leprosy strains was much higher than that of SNP type 1 (*x* ^2^ = 35.017, *p*<0.01). The relationship between strains with and without C251T mutation for type 3 strains with different pathology types was also analyzed. Among the 54 strains with the C251T mutation, 45 caused multibacillary leprosy and among 13 wild type strains, 9 caused multibacillary leprosy. This indicated that there is no difference between the C251T mutation and clinical type.

**Table 3:** Comparison between 16S rRNA gene mutation and SNP genotype according to geographic distribution in China (n = 85).

Region		Province	SNP genotype	Number	16S rRNA gene mutation	
					C251T	%
Coastal	East	Shandong	3	3	2	66.7
		Jiangsu	3	7	7	100
		Zhejiang	3	1	1	100
		Fujian	1	4	0	0
			3	7	3	42.9
		Shanghai	3	2	2	100
	South	Hainan	3	1	0	0
		Guangdong	1	12	1	8.3
			3	9	5	55.6
		Guangxi	1	1	0	0
			3	3	1	33.3
Inner	Central	Anhui	3	4	4	100
		Hunan	3	6	6	100
		Jiangxi	3	2	1	50
	Northwest	Shanxi	3	1	1	100
		Gansu	3	3	3	100
		Xinjiang	2	1	0	0
	Southwest	Tibet	3	1	1	100
		Sichuan	3	7	7	100
		Guizhou	3	3	3	100
		Yunnan	3	7	7	100
	Total		1	17	1	5.9
			3	67	54	80.6
			2	1	0	0
	Statistical tests	*x* ^*2*^	33.707			
		*p*	0.000			

### Comparative analysis of 16S rRNA gene mutation and SNP genotype in patient samples from different regions

We compared the distribution of different SNP genotypes in samples from different regions in China and found that SNP type 1 leprosy strains are located only in the coastal region (Table 4). Whereas, the SNP type 2 and SNP type 3 strains coexisted in the interior regions of the country. However, SNP type 3 strains were found in both the coastal region and the inner parts the country. Apparently, the SNP type 3 strains were more prevalent than the SNP type 1 strains in China. There was a significant difference in distribution for the SNP genotypes from different regions (*x* ^2^ = 43.29, *p* < 0.01). In addition, the mutation frequency of the 16S rRNA gene of leprosy strains from the interiors was higher than that of the strains from the coastal region (Table 4). There was also a significant difference between the 16S rRNA gene sequence and geographic distribution (*x* ^2^ = 17.695, *p* < 0.05) (Table 4).

**Table 4:** Comparison of 16S rRNA gene sequence and SNP genotype of strains from different regions.

Region		Province	16S rRNA gene mutation			SNP genotype			
			Number	C251T	%	Number	1	2	3
Coastal	East	Shandong	5	4	80	3			3
		Jiangsu	7	7	100	7			7
		Zhejiang	2	2	100	1			1
		Fujian	15	6	40	11	4		7
		Shanghai	2	2	100	2			2
	Northeast	Liaoning	1	1	100				
		Jilin	1	0	0				
	South	Hainan	4	2	50	1			1
		Guangdong	31	11	35.5	21	12		9
		Guangxi	6	3	50	4	1		3
Inner	Central	Anhui	4	4	100	4			4
		Hubei	2	2	100				
		Hunan	6	6	100	6			6
		Jiangxi	2	1	50	2			2
	North	Beijing	1	1	100				
		Shanxi	1	1	100	1			1
		Xinjiang	1	0	0	1		1	
		Gansu	3	3	100	3			3
	Southwest	Sichuan	24	23	95.8	7			7
		Yunnan	27	27	100	7			7
		Guizhou	24	23	95.8	3			3
		Tibet	2	1	50	1			1
Coastal region			74	38	50.66	50	17		33
Inner region			97	92	93.94	35	0	1	34
Total			171	130	75.57	85	17	1	67
Statistical tests			*x* ^*2*^	43.564		18.330			
			*p*	0.000		0.000			

Only SNP type 3 leprosy strains were found in the inner provinces and had the mutant 16S rRNA gene. However, SNP type 1 leprosy strains did not have the 16S rRNA mutation and were found in the coastal region.

## Discussion

A total of 171 *M*. *leprae* samples were collected from 22 provinces in China where there were still reports of leprosy cases. Thus, these strains represented the epidemiological status of leprosy in China. However, only 85 samples could be genotyped due to a lack of DNA samples. Nevertheless, analyzing these samples helped us draw some conclusions about the molecular epidemiology of the leprosy strains in China. Our analysis indicated that three genotypes, SNP type 1, SNP type 2, and SNP type 3 exist in China, while SNP type 4 strains were not found. Most of the strains belonged to SNP type 3, accounting for about 78.8% (67/85) of samples, and were distributed over both inner and coastal areas of China. Furthermore, some strains belonged to SNP type 1, and accounted for 19.3% (17/88) of the samples. These strains were found only in the southeast coastal regions of China such as Guangdong, Guangxi, and the Fujian province. Additionally, 1 leprosy strain from the Xinjiang municipality belonged to SNP type 2. Our genotyping study as well as earlier reports on leprosy strains indicate that only three genotypes are found in China [14, 15] whereas, there are four genotypes in the world [4, 16]. According to previous research, the genotype of leprosy strains in Europe, northern Africa, Southern America, central Asia, Turkey, Iran as well as Japan, Korea, Indonesia, and Philippines belong to SNP type 3 [17–19]; the genotype of leprosy strains in India, Thailand, and Nepal belong to SNP type 1 [20, 21]; and the genotype of strains in Burma and East Africa belong to SNP type 2 [22]; SNP type 4 leprosy strains exist only in western Africa and countries linked to West Africa by the slave trade [4, 16]. The SNP type 3 and SNP type 2 leprosy strains are the most dominant strains in the world.

Compared with the genotypes of leprosy strains from neighboring Asian countries and other parts of the world, there are three possible routes of leprosy transmission to China. The first is the northern route via the Silk Road from Central Asia which was used during the Han Dynasty (202 BC–220 AD). This could be a possible route because the leprosy strains identified in the countries along the route belong to SNP type 3 [17–19], and are similar to the genotype transmitted to inner southwestern provinces of China such as Yunnan, Sichuan, and Guizhou. The second is through the southern Silk Road on the sea, which involved countries of Southeast Asia and was influential during the Tang Dynasty in the 8^th^ century. This route was responsible for the spread of dominant SNP type 1 strains to countries such as Thailand, Philippines, India, and Nepal [20, 21]. These strains are identical to the genotype of strains from southern provinces of China such as Guangdong, Guangxi, and Fujian. The third is the eastern route, which is another marine Silk Road bridging China to Japan and Korea via the eastern sea from Ningbo city in the Zhejiang province. The genotype of leprosy strains from these countries is the same as that of strains from eastern coastal provinces such as Zhejiang, Jiangsu, and Shanghai. [17–19].

One major goal of our study was to confirm the existence of the novel leprosy species *M*. *lepromatosis* in China. However, we did not detect the species in the samples we analyzed. Using the available data of SNP genotypes, we compared the characteristic 16S rRNA gene sequence of the strains with known SNP genotypes. Interestingly, we found that there was a link between the mutations in the 16S rRNA gene sequence and SNP type. Most of the strains with the variant 16S rRNA gene, C251T belong to SNP type 3. The strains without this mutation belong to SNP type 1 and SNP type 2. Furthermore, we compared the geographic distribution of these strains to mutations in the 16S rRNA gene sequence and only strains without the mutation exist in the coastal provinces of China. The strains with the 16S rRNA gene C251T mutation exist in both the inner and coastal provinces. The geographic distribution for 16S rRNA gene sequence is similar to that of the SNP genotype. The strains with the 16S rRNA gene C251T mutation seem to have a more widespread distribution than those without the mutation. It is unclear why strains with this C251T mutation are so prevalent in China. It is possible that such strains are more fit and easier to transmit than other strains. In support of the latter possibility, there is evidence to suggest that this type of leprosy strains are still spreading in southeast area of China including Yunnan, Guizhou, and Sichuan provinces where there is a higher prevalence of the disease [23]. The finding that strains without the 16S rRNA gene C251T mutation exist in the area where there is a lower prevalence of the disease [24] also suggests that such strains are much easier to transmit among a population than other strains. Future studies are needed to assess if the dominant strain can transmit in humans more readily. In addition, further investigation of the genomes of these strains, with possible phylogenetic analysis comparing the genomes will also shed light on the genome structure of these strains.

Besides the association with high transmission, the biological function of the mutation in the 16S rRNA is unclear. It is well known that mutations in 16S rRNA in *M*. *tuberculosis* can lead to resistance to aminoglycoside antibiotics [25]. Similarly, whether the mutations in 16S rRNA in *M*. *leprae* are also associated with resistance to anti-leprosy drugs can only be determined by further studies.

In summary, we characterized the genotypes of leprosy strains in China and identified SNP type 3 as the most prevalent genotype in China. We also found a new polymorphism C251T in the 16S rRNA gene that is primarily associated with SNP type 3 leprosy strains in China. Future studies will determine if this mutation is related to antibiotic resistance. Finally, the new species of *M*. *lepromatosis* was not found in leprosy patients in China. Our findings provide new insight into the genotype structures of leprosy strains in China and improve our understanding of the transmission and molecular epidemiology of leprosy which are useful for leprosy control in China.
